# Multiscale Computational Studies of PEG Chain Length Effects on HER2 mAb Fc Structure and Binding Energetics

**DOI:** 10.1021/acsomega.5c08654

**Published:** 2025-12-05

**Authors:** Heather A. Noriega, Emmanuel O. Akala, Xiang Simon Wang

**Affiliations:** † Artificial Intelligence and Drug Discovery (AIDD), Core Laboratory for District of Columbia Center of AIDS Research (DC CFAR), Washington, D.C. 20052, United States; ‡ Department of Pharmaceutical Sciences, College of Pharmacy, Howard University, Washington, D.C. 20059, United States

## Abstract

HER2 overexpression in breast cancer drives aggressive disease, treated clinically with monoclonal antibodies such as trastuzumab and pertuzumab. While effective, these therapies are limited by suboptimal pharmacokinetics and tumor penetration. Polyethylene glycol (PEG) conjugation can extend circulation half-life but may alter Fc-mediated interactions and receptor binding. Here, we used a multiscale computational framework to quantify PEGylation effects on the pertuzumab Fc domain. Structural models were generated with AlphaFold2, refined with RoseTTAFold2 to incorporate G0F-type glycans at Asn297, and site-specifically PEGylated via hydrazone linkages in UCSF ChimeraX. Aglycosylated control and PEGylated variants (1, 2, and 4 kDa) underwent 100 ns all-atom molecular dynamics simulations in GROMACS. Increasing PEG size produced stepwise RMSD elevations (1.1–13 nm) and hinge expansion (21.37–63.53 Å). RMSF analysis revealed domain-specific mobility shifts within the Fc region: CH2 flexibility in the control, CH3 mobility in the 1 and 2 kDa variants, and CH2/CH3 destabilization (>2.0 nm) in the 4 kDa system. Principal component analysis showed PC1 (45–70% variance) capturing hinge-closing and CH2 inward motion in controls, versus CH3 separation and CH2–CH3 displacement in PEGylated forms; the 4 kDa variant exhibited pronounced flexibility (PC2 ∼20 to 25%). Complementary backbone dihedral and hydrogen bond analyses showed localized torsional relaxation and a reduction in CH2–CH3 hydrogen bond occupancy in PEGylated systems, confirming the structural basis of hinge expansion. Vector projections indicated steric and entropic disruption of interdomain hydrogen bonds, suggesting reduced Fcγ receptor engagement. These results reveal PEG size-dependent structural perturbations, providing a molecular basis for diminished HER2 affinity and guiding rational design of PEGylated mAbs with optimized pharmacokinetics and preserved effector function.

## Graphical abstract

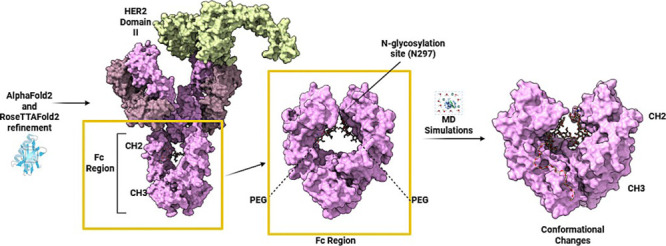

## Introduction

Human epidermal growth factor receptor 2 (HER2/ERBB2) is a member of the ERBB receptor tyrosine kinase family and plays a key role in regulating cell proliferation, survival, and differentiation. Unlike other ERBB family members, HER2 lacks a known ligand. It adopts a constitutively open conformation that readily forms homodimers or heterodimers with other receptors such as EGFR (ERBB1) and HER3 (ERBB3), leading to activation of downstream signaling cascades including the PI3K/AKT and MAPK pathways.^1,2^ Amplification or overexpression of HER2 occurs in approximately 15–20% of breast cancers and is associated with increased tumor aggressiveness, higher recurrence rates, and reduced overall survival.^3,4^ The development of HER2-targeted monoclonal antibodies (mAbs) has significantly improved the outcomes in this patient population.^5,6^

Two clinically approved mAbs, Trastuzumab and Pertuzumab, target distinct epitopes on the extracellular domain of HER2. Trastuzumab binds to subdomain IV, near the transmembrane region, inhibiting ligand-independent signaling and promoting antibody-dependent cellular cytotoxicity.^7,8^ Pertuzumab binds to subdomain II, the dimerization arm, sterically blocking heterodimerization, most notably HER2:HER3, a highly oncogenic complex.^9^ Combined, these agents provide complementary inhibition, translating into clinically meaningful benefits: in the metastatic setting, the CLEOPATRA trial demonstrated significant gains in progression-free and overall survival with Pertuzumab + Trastuzumab + docetaxel versus Trastuzumab + docetaxel alone;^10^ in the neoadjuvant setting, NeoSphere showed higher pathological complete response rates with dual HER2 blockade;^11^ and in early, high-risk node-positive disease, APHINITY reported improved invasive disease-free survival with adjuvant Pertuzumab added to Trastuzumab and chemotherapy.^12^ These data establish dual HER2 targeting as a standard-of-care strategy across disease stages and underscore the importance of preserving epitope accessibility and binding efficacy when engineering Fc-modified or PEGylated antibody formats.^13^

Despite clinical efficacy, therapeutic monoclonal antibodies face pharmacokinetic and delivery challenges. Systemic clearance, tumor penetration, and immune system interactions can limit bioavailability at the tumor site. Polyethylene glycol (PEG) conjugation is a commonly employed strategy to extend circulation time and reduce immunogenicity by increasing hydrodynamic size and shielding epitopes from immune surveillance.^14,15^ PEGylation of antibodies is commonly performed at or near the Fc region, where it can influence not only pharmacokinetics but also the structural conformation and receptor engagement. The Fc domain consists of two heavy-chain constant domains: CH2 and CH3. The CH2 domain includes a conserved N-linked glycosylation site at Asn297, which is essential for interactions with Fcγ receptors and the neonatal Fc receptor (FcRn), both of which mediate effector function and prolong circulation time.^16,17^ This domain also provides the flexibility required for immune activation. In contrast, the CH3 domain forms the dimerization interface between antibody heavy chains and contributes primarily to Fc stability and structural integrity.^18,19^ These regions are shown in Figure 1. The CH2 and CH3 domains exhibit distinct but coordinated conformational behavior, and their spatial orientation plays a critical role in Fc receptor binding and effector function. Molecular dynamics (MD) simulations have shown that these domains are structurally flexible and responsive to environmental perturbations, including hinge bending and domain displacement.^20,21^ Although PEGylation was not directly studied, such conformational sensitivity suggests that covalent modifications or adjacent steric bulk, such as PEG chains, could alter domain positioning and reduce accessibility to functional binding sites, as hypothesized in prior reviews.^14,22^

**Figure 1:**
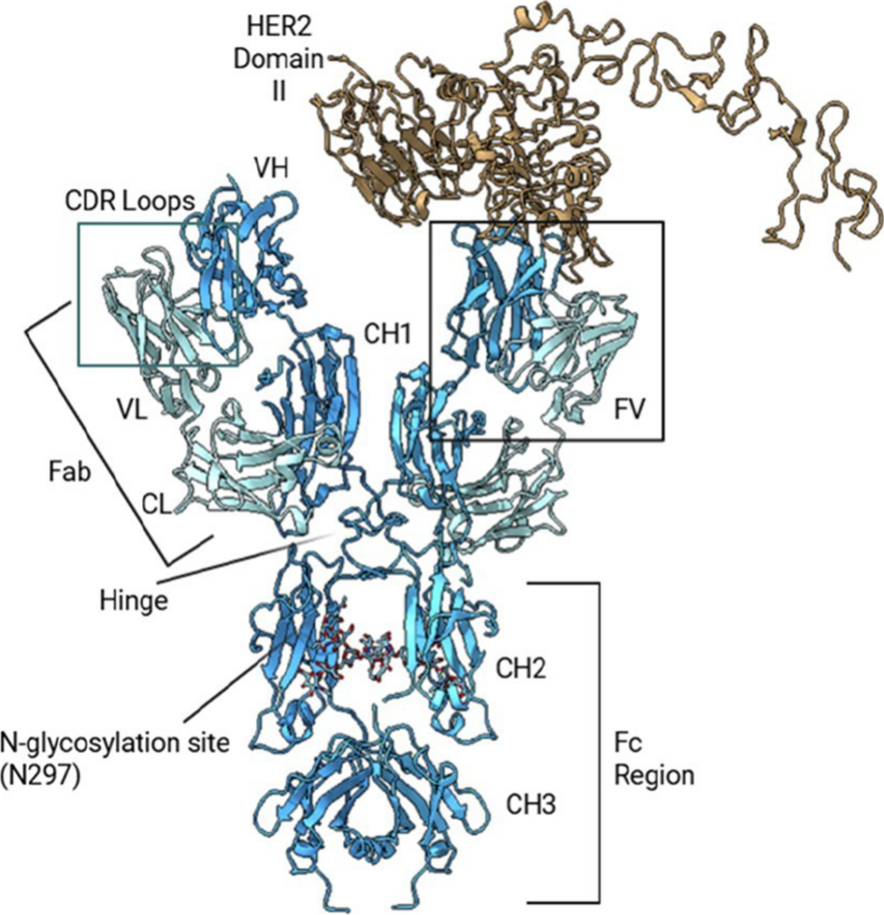
Structure of Pertuzumab bound to HER2 domain II. The IgG1 antibody Pertuzumab is shown in complex with HER2 domain II (brown). Key regions are labeled, including the Fab arms, CDR loops, hinge, Fc region (CH2/CH3), and N-glycosylation site at N297. Pertuzumab binds HER2 at subdomain II to block receptor dimerization.

Experimental data have shown that the PEG linker length directly influences receptor binding and bioactivity in PEGylated antibody and antibody fragment systems. A recent study by Li et al. demonstrated that inserting 4 and 10 kDa PEG linkers into HER2-targeted drug conjugates progressively reduced cytotoxic activity in vitro, by approximately 4.5-fold and 22-fold, respectively, due to steric interference with receptor binding and internalization, despite improved circulation times.^23^ Another example, in 2020, Fisusi et al. reported that Pertuzumab modified with 2 kDa PEG (2K-pMAb) showed a reduction in HER2 binding affinity and cytotoxic efficacy compared to the non-PEGylated antibody.^24^ In 2016, Selis et al. study evaluated single-chain Trastuzumab fragments conjugated with 10 and 20 kDa PEGs and observed progressive losses in antigen-binding capacity, with 20 kDa PEGylation causing the most severe impairment due to steric hindrance and conformational restriction.^25^ Furthermore, Kubetzko et al. systematically investigated the effects of PEGylation and multimerization on a HER2-specific single-chain antibody fragment (scFv 4D5). By comparing unmodified monomers, dimeric and tetrameric mini-antibodies, and site-specifically PEGylated constructs, they demonstrated that PEGylation caused a ∼5-fold reduction in binding affinity, primarily due to reduced association rates, despite the PEG site being distal from the antigen-binding domain. In contrast, multimerization increased the functional affinity via avidity effects. Importantly, the PEGylated monomer and dimer showed 8.5-fold and 14.5-fold increases in tumor accumulation, respectively, due to prolonged serum half-life, highlighting the trade-off between binding strength and in vivo pharmacokinetics. This work underscores that PEGylation can preserve immunoreactivity while significantly altering kinetic binding behavior, and that linker size and format selection must consider both tumor localization and receptor engagement dynamics.^26,27^

Together, these studies support the hypothesis that the PEG size imposes a length-dependent effect on antibody performance. While PEGylation enhances pharmacokinetic properties, it also introduces a structural penalty that may involve altered CH2/CH3 domain motion or hinge flexibility, mechanisms not yet fully elucidated at atomic resolution. Understanding how PEG chains of increasing length perturb the Fc conformation is critical to optimizing linker design for therapeutic antibodies and conjugates.

Computational methods offer a mechanistic framework to examine these effects at an atomic resolution. Recent advances in deep-learning-based protein structure prediction have enabled highly accurate modeling of antibody architectures. AlphaFold2 and RoseTTAFold2 have demonstrated high-resolution performance in predicting the structures of full-length antibodies, including variable and constant domains, by leveraging coevolutionary signals and attention-based neural networks.^28,29^ These models have proven particularly useful for generating structural hypotheses when experimental data are limited, especially for regions such as the Fc domain and CH2/CH3 subdomains, which are sensitive to post-translational modifications and conjugation.

These developments have recently progressed further with the introduction of AlphaFold3, which expands predictive capabilities to include protein–nucleic acid and protein–ligand interactions and offers support for simple glycan structures.^30^ Although AlphaFold3 does not yet model PEGylated constructs, its underlying framework reflects an increasing capacity to handle multicomponent biological assemblies, suggesting potential applicability to PEG–protein conjugates in future versions.

To translate predicted antibody structures into functionally analyzable systems, molecular modeling platforms such as UCSF ChimeraX are widely used. ChimeraX enables the integration of post-translational modifications, manual conjugation of polymers like PEG, and structural alignment to experimental reference maps.^31^ Refined models are often validated using high-resolution data from repositories such as the Protein Data Bank (PDB) and the Electron Microscopy Data Bank (EMDB), which provide established structural frameworks for IgG1 Fc regions and associated glycan moieties.^32–34^

Following structural assembly, MD simulations are performed to characterize time-resolved atomic motions and domain-specific flexibility under physiological conditions. MD has been applied extensively in immunoglobulin research to explore Fab–Fc transitions, CH2–CH3 interface stability, glycosylation-induced mobility, and hinge-bending phenomena.^35,36^ For PEGylated constructs, MD can capture steric hindrance effects and spatial dislocation of the Fc region, potentially affecting receptor interactions or effector recruitment.^37^

To quantify large-scale motions from MD trajectories, principal component analysis (PCA) is applied. PCA reduces complex conformational data into orthogonal eigenvectors representing dominant motions. In the context of Fc dynamics, PCA has been used to map CH2/CH3 displacement, hinge flexibility, and allosteric coupling between Fc and Fab domains.^38–41^ Such analyses provide insight into structural perturbations that may not be readily apparent in static models and are increasingly used to interpret the mechanistic consequences of modifications, such as glycoengineering.

Despite substantial progress in structural prediction and dynamic modeling, the mechanistic basis by which PEGylation influences the Fc-region flexibility and receptor binding remains insufficiently defined. In particular, how variations in PEG linker length modulate CH2/CH3 mobility, hinge conformation, and long-range domain coupling has not been resolved at atomic resolution. Experimental studies have reported decreased HER2-binding affinity in PEGylated HER2 antibody constructs, particularly at PEG sizes ≥ 2 kDa, yet the structural correlates underlying this functional loss remain elusive. As PEGylation is increasingly utilized in antibody–nanoparticle conjugates, a detailed understanding of these conformational effects is needed to inform rational linker design. The following computational investigation addresses this gap by evaluating the structural dynamics, essential motions, and energetic properties of Fc-PEG conjugates with varying linker lengths using multiscale AI/ML and computational simulation techniques.

## Methods

### Structural Modeling and System Preparation

Four systems were constructed: (1) a non-PEGylated with no glycan Pertuzumab Fc control and (2–4) three PEGylated Pertuzumab Fc conjugates with (2) 1 kDa ≈ 22 monomers; 2 kDa ≈ 45; 4 kDa ≈ 91 linear PEG linkers, respectively. The base structure of Pertuzumab Fc was predicted using AlphaFold2 and refined using RoseTTAFold2. UCSF ChimeraX was used to assemble full models, incorporating G0F-type core-fucosylated glycans at the conserved Asn297 site, consistent with known Trastuzumab and IgG1 glycoforms based on PDB structures and batch-to-batch profiling done by Waters Corporation.^34,42,43^

PEGylation was modeled using a chemical conjugation strategy adapted from Fisusi et al., which enables the covalent linkage of PEG chains to terminal sugar moieties of Fc-associated glycans. Specifically, the terminal N-acetylglucosamine (NAG) on each glycan was targeted for chemical oxidation. In experimental settings, sodium periodate (NaIO_4_) was used to oxidize the C6 primary hydroxyl group of NAG, generating a reactive aldehyde intermediate. This electrophilic site was then conjugated to a hydrazide-functionalized poly­(ethylene glycol) monomethacrylate (PEGMMA) via hydrazone formation, yielding a stable covalent linkage.^24^ The resulting PEGylated antibodies were purified by HPLC and characterized to confirm the site-specific attachment to the Fc domain.

In ChimeraX, this reaction was mimicked manually by positioning the hydrazide group of the PEGMMA chain in proximity to the aldehyde group generated on the C6 carbon of oxidized N-acetylglucosamine (NAG). The covalent hydrazone linkage was then formed between the nitrogen of the hydrazide and the carbonyl carbon of the NAG aldehyde group. Bond lengths and angles were adjusted to match typical hydrazone geometry, and local energy minimization was applied to relieve steric clashes and optimize conformation. This approach ensured topological continuity among the Fc, glycan, and PEG units, yielding chemically and structurally realistic starting conformations for downstream simulations.

### Structural Model Validation

The structural quality of the four Pertuzumab Fc constructs, including a non-PEGylated without glycan control and PEGylated forms with 1, 2, and 4 kDa PEG linkers, was evaluated using a combination of deep-learning–based prediction metrics, experimental structural alignments, and topological assessments. Structural modeling was initiated with AlphaFold2, which provides key confidence scores: predicted Local Distance Difference Test (pLDDT) and predicted Template Modeling score (pTM).

The pLDDT is a residue-level measure ranging from 0 to 100, indicating the confidence of backbone prediction. All four Fc constructs exhibited pLDDT values above 70 across their sequences, reflecting highly confident local structure predictions. The pTM score, which evaluates global domain orientation on a 0–1 scale, exceeded 0.7 for all models, indicating reliable interdomain and dimeric organization, particularly at the CH3 dimer interface and hinge-proximal CH2 regions.

Refinement of the AlphaFold2 models was performed using RoseTTAFold2, which improved the loop geometry and side-chain packing, especially around the lower hinge and N-glycosylation site at Asn297. Structural alignment with experimentally resolved human IgG1 Fc fragments (PDB: 5VGP and 3AVE) showed backbone RMSD values below 1.0 nm for all systems.

Predicted Alignment Error (PAE) matrices provided an additional layer of validation. The PAE matrix estimates the expected distance error between residue pairs following optimal alignment. Low predicted errors (<5 Å) were observed throughout the CH2 and CH3 domains, indicating high confidence in domain topology. As expected, the hinge region showed greater uncertainty, reflecting its intrinsic flexibility. Notably, the PAE maps confirmed well-ordered interdomain orientations across all constructs.

Fitting backbone atomic coordinates further verified the model geometry and topology to cryo-EM density maps of human IgG1 Fc fragments available in the Electron Microscopy Data Bank (EMDB). Fit-to-map scoring confirmed a high degree of concordance (score >0.7) between predicted structures and resolved density in the CH2–CH3 core, supporting the validity of the predicted Fc fold.

Glycosylation at the conserved Asn297 site was modeled by incorporating the predominant G0F-type biantennary N-linked glycan, consistent with prior profiling of Trastuzumab glycoforms and human IgG1 Fc glycoproteins. While the cryo-EM maps were not used to directly model glycans, their inclusion was structurally validated by overlaying with glycosylated Fc structures in PDB entries and adjusted in UCSF ChimeraX to minimize steric clashes and maintain canonical glycosidic linkage geometry.

### MD Simulations

All-atom MD simulations were performed for each of the four systems using GROMACS 2022.4.^44^ Protein and glycan components were parametrized using the CHARMM36m force field,^45^ while PEG topologies were generated using the CHARMM General Force Field (CGenFF).^46^ Each model was solvated in a cubic water box using TIP3P water molecules with a minimum of 1.0 nm buffer, neutralized with counterions, and brought to physiological ionic strength (150 mM NaCl).

Energy minimization was conducted using the steepest descent algorithm, followed by position-restrained equilibration in both the NVT and NPT ensembles. Production runs were carried out for 100 ns per system in the NPT ensemble at 300 K and 1 atm using a velocity rescaling thermostat and Parrinello–Rahman barostat. Long-range electrostatics were handled by using the particle mesh Ewald (PME) method with a 1.2 nm cutoff for Coulomb and van der Waals interactions. All bonds involving hydrogen were constrained using LINCS, enabling a 2 fs integration time step.

### Structural Analysis and Trajectory Evaluation

Trajectory frames were saved every 10 ps and analyzed by using GROMACS built-in tools and custom Python scripts. Structural stability was assessed using root-mean-square deviation (RMSD) for backbone atoms as well as the glycan and PEG moieties. Root mean square fluctuation (RMSF) was calculated per residue representing the Fc heavy chain, encompassing both the CH2 and CH3 domains.

Conformational displacements were assessed by measuring the inter-residue distance between selected hinge-proximal residues. These residues, positioned symmetrically on opposing chains, were monitored throughout the trajectory to track changes in hinge opening or closing. Global motion was further analyzed using PCA on all protein residues, as implemented in the GROMACS anaeig module. Eigenvectors were extracted from the full covariance matrix of atomic positional fluctuations, capturing the dominant collective motions of the entire Fc structure. The first three principal components (PC1–PC3) were retained to assess large-scale conformational dynamics, including hinge flexibility and CH2/CH3 domain displacements, and to evaluate how these global motions were affected by PEGylation with varying linker lengths.

Backbone dihedral (φ/ψ) distributions were computed using GROMACS rama and angle to assess local conformational sampling across each Fc construct. Hydrogen bond persistence and count were obtained using the GROMACS hbond with a donor–acceptor distance cutoff of 0.35 nm and an angle cutoff of 30°. All analyses were conducted on equilibrated trajectories following the RMSD stabilization. Temporal profiles and averaged values were calculated over the 100 ns production runs to identify changes in interdomain contacts and backbone flexibility associated with PEGylation.

## Results

### Structural Dynamics and PCA

All-atom MD simulations demonstrated robust thermal stability across all systems (Figure 2A), maintaining an average potential energy of −30,000 kJ/mol throughout the 100 ns production runs. (Figure 2B) The non-PEGylated control system rapidly stabilized, with the RMSD plateauing at approximately 1.1 nm within the initial simulation phase, reflecting minimal structural perturbation (Figure 2C). In contrast, PEGylated systems exhibited progressive increases in the RMSD correlated with increasing PEG linker length. Specifically, the 1 kDa PEGylated system reached a plateau RMSD of ∼2.1 nm; the 2 kDa system stabilized at ∼4.5 nm, and the 4 kDa system displayed the highest deviation, peaking at ∼13 nm. These trends suggest that longer PEG chains induce greater structural flexibility and deviation from the initial conformation, highlighting the impact of the extent of PEGylation on the dynamic behavior of the pertuzumab (Pmab) glycan conjugates. Because the RMSD was calculated including both glycans and PEG linkers, part of this increase reflects the intrinsic mobility of PEG; however, the consistency of RMSF backbone profiles across systems confirms that PEG length also contributes to Fc domain flexibility.

**Figure 2:**
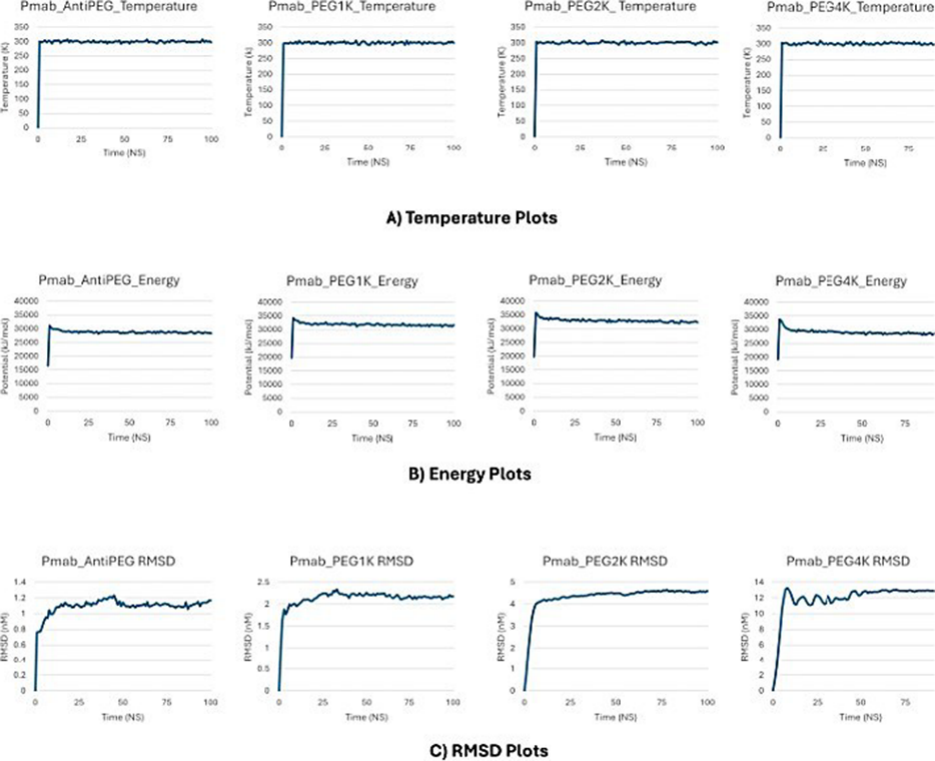
System equilibration profiles for non-PEGylated and PEGylated Pertuzumab Fc constructs. (A) *Temperature plots* showing stable thermal equilibration near 300 K across all systems throughout 100 ns simulations. (B) *Energy plots* indicate rapid convergence of potential energy within the first few nanoseconds, confirming the thermodynamic stability of each trajectory. (C) *RMSD plots* demonstrate initial structural relaxation within the first 10–20 ns, followed by stable fluctuations, with increasing RMSD magnitudes observed for longer PEG chains (1 kDa < 2 kDa < 4 kDa), consistent with enhanced conformational flexibility upon PEGylation.

Inter-residue distance measurements between conserved hinge-proximal residues revealed a compacting trend in the non-PEG and glycan control system, decreasing from 29.77 to 21.37 Å over the trajectory. This closure likely reflects the stabilizing influence of native Fc-associated glycans, which mediate intradomain hydrogen bonding and hydrophobic interactions to maintain a compact hinge conformation. In contrast, PEGylated variants showed progressive hinge expansion, with distances increasing to 35.34 Å (1 kDa), 53.40 Å (2 kDa), and 63.53 Å (4 kDa), indicating that PEG linker length correlates with enhanced Fc domain separation and hinge opening (Figure 3). The importance of glycans in this context lies in their role in optimizing effector functions such as Fcγ receptor binding and antibody half-life, which the steric hindrance introduced by PEGylation may disrupt. RMSF values were computed for residues 1–422, corresponding to the Fc heavy chain containing both CH2 and CH3 domains. For clarity, RMSF values from both heavy chains have been overlaid within each graph, producing a single combined trace that reflects the average per-residue fluctuations across the Fc dimer. Only one CH2 and CH3 region is labeled to represent the symmetric motion of both chains and to minimize visual redundancy. As illustrated in Figure 4, RMSF profiles revealed distinct patterns in magnitude and localization across the non-PEGylated control (lacking glycans) and the 1, 2, and 4 kDa PEGylated variants of pertuzumab (Pmab). The non-PEGylated control, devoid of glycan stabilization, exhibited elevated flexibility with RMSF values peaking.

**Figure 3:**
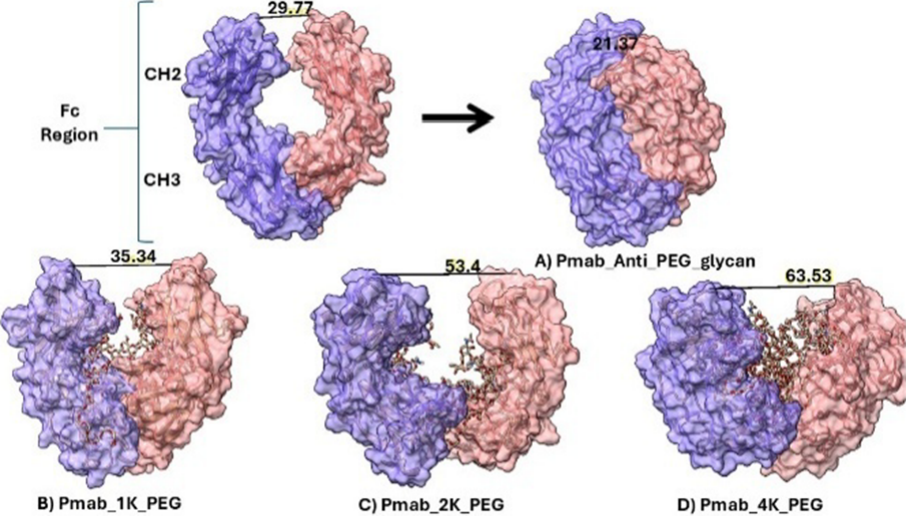
Structural dynamics of the pertuzumab (Pmab) Fc region upon PEGylation. Molecular simulations illustrate the anti-PEGylated glycan control (A) with a hinge-proximal inter-residue distance decreasing from 29.77 to 21.37 Å and PEGylated variants with distances of 35.34 Å (B, 1 kDa), 53.40 Å (C, 2 kDa), and 63.53 Å (D, 4 kDa) at the end of 100 ns, highlighting progressive hinge expansion with increasing PEG linker length. The pink (heavy chain 1) and purple (heavy chain 2) surfaces represent Fc heavy chains, respectively, corresponding to the CH2–CH3 domains of the Fc region shown in the start figure.

**Figure 4:**
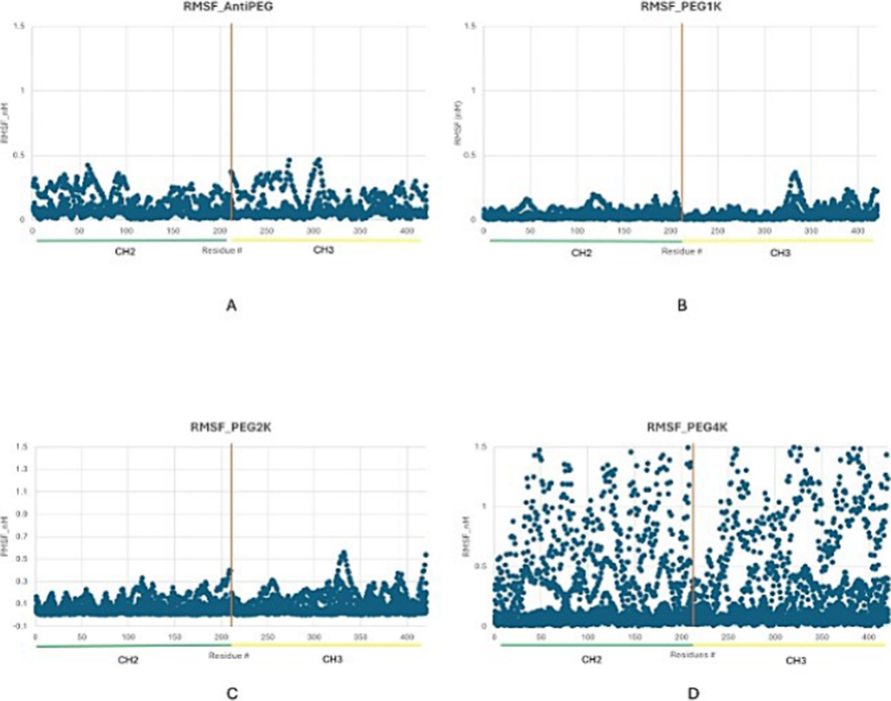
Root mean square fluctuation profiles of the Fc region across PEGylated and non-PEGylated systems: (A) anti-PEG control, (B) PEG-1 kDa, (C) PEG-2 kDa, and (D) PEG-4 kDa. Each plot shows residue-wise flexibility (RMSF, Å) along the Fc domain, with CH2 and CH3 domains labeled below the *x*-axis and the orange vertical line denoting the hinge interface. Increasing PEG length corresponded to a progressive redistribution of flexibility from the CH2 region toward CH3, with the 4 kDa construct exhibiting the highest overall amplitude.

Across all systems, RMSF magnitudes remained below 0.5 nm in the anti-PEGylated construct with peaks concentrated in the CH2 region near the hinge. The 1 kDa variant and 2 kDa displayed attenuated CH2 motion and emerging CH3-domain flexibility, while the 4 kDa construct exhibited progressive amplification of CH2/CH3 fluctuations and global destabilization. This pattern indicates a shift in intrachain flexibility from CH2-localized motion in the control toward CH3-centered dynamics as the PEG length increases. These findings highlight a PEG length-dependent redistribution of flexibility, with the aglycosylated non-PEGylated control showing dominant CH2 mobility, whereas PEGylation progressively enhances CH3-domain motion, culminating in widespread destabilization in the 4 kDa variant. This trend aligns with PCA results and suggests altered domain interactions that may influence receptor binding efficiency due to increased structural disorder.

PCA was performed on the Cα backbone atoms using an eigenbasis computed from the concatenated, backbone-aligned trajectories of all systems (Figure 5). Principal component (PC1) captured 45–70% of the total variance. The non-PEGylated control lacking. The purpose of this study was to investigate how covalent PEGylation at the Fc glycan region influences the structural dynamics and binding energetics of HER2-targeted monoclonal antibodies. modifications, exhibited a PC1 variance of approximately 45–50%, reflecting a constrained motion landscape dominated by hinge-closing and inward tilting of the CH2 domain, as evidenced by the compact vector field projection. This behavior aligns with reports that N297 glycans stabilize CH2 packing, in hinge-present systems, removal of glycans lowers free-energy cost for both CH2 closure at short separations and CH2–CH2 separation relative to glycan-present systems.^20^ This motion likely arises from the absence of glycan-mediated stabilization, allowing intrinsic flexibility in the CH2 region to manifest as a low-amplitude, collective movement, with an estimated contribution from PC2 of less than 15%, indicative of limited secondary conformational diversity. In contrast, PEGylated variants displayed a progressive increase in PC1 variance with linker length: the 1 kDa variant accounted for ∼50 to 55% variance with initial CH3 domain separation, the 2 kDa variant reached ∼55 to 60% with enhanced outward CH3 motion, and the 4 kDa variant peaked at ∼60 to 70%, accompanied by a notable PC2 contribution (∼20 to 25%), reflecting a broader conformational ensemble. The vector field projections for PEGylated systems revealed a transition from localized CH3 displacements in the 1 kDa variant to extensive CH2–CH3 splaying and torsional mobility in the 4 kDa variant, with vector magnitudes increasing from ∼0.5 to >2.0 Å, particularly near the glycan-PEG junction (residues 290–310). This suggests that PEGylation introduces steric and entropic penalties, disrupting native interdomain hydrogen bonding networks (e.g., CH2–CH3 interface contacts) and promoting a dose-dependent expansion of the Fc elbow angle, potentially ranging from 90° to 120° based on typical IgG hinge geometries. To further validate this structural interpretation, backbone dihedral (φ/ψ) and hydrogen bond analyses were performed. Ramachandran density plots show that PEGylated variants exhibited broader φ/ψ sampling localized to residues 320–340 within the CH2–CH3 interface, consistent with torsional relaxation and hinge opening. Complementary hydrogen bond analysis demonstrated a reduction in interdomain hydrogen bond occupancy relative to the anti-PEG control, particularly at the CH2–CH3 interface, confirming that PEGylation weakens stabilizing contacts and facilitates hinge expansion (Figure 6).

**Figure 5:**
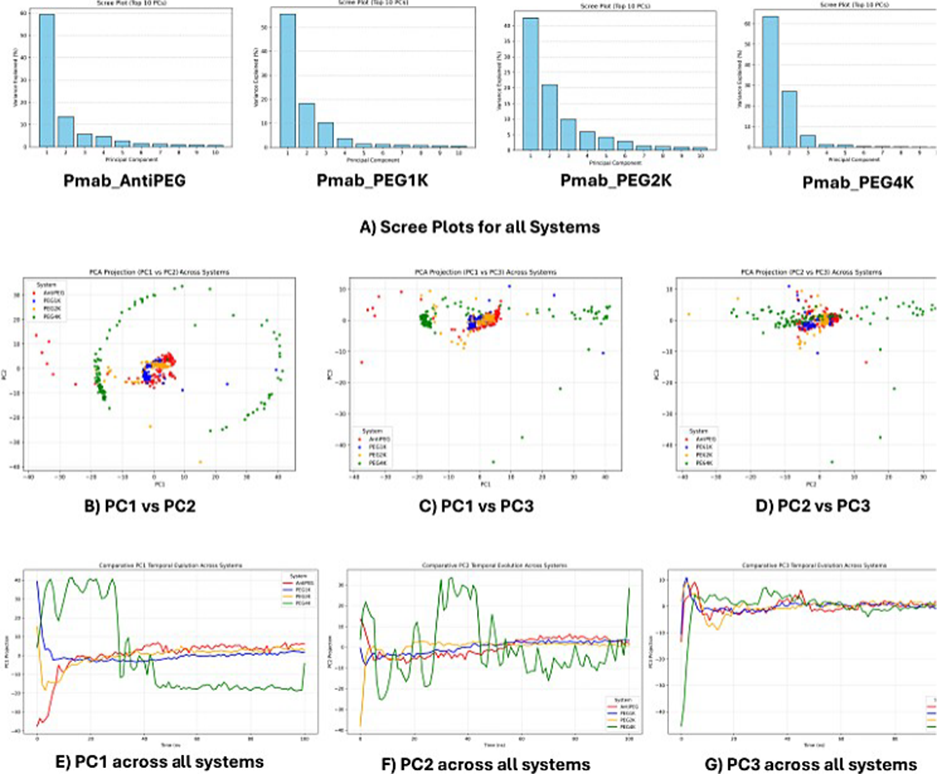
Principal component analysis (PCA) of pertuzumab (Pmab) Fc constructs. (A) Scree plots showing eigenvalue distribution for each system (AntiPEG, PEG1K, PEG2K, and PEG4K). (B–D) 2D projections of principal components illustrating conformational clustering and motion along PC1-PC2, PC1-PC3, and PC2-PC3. (E–G) Temporal evolution of PC1, PC2, and PC3 across all systems.

**Figure 6:**
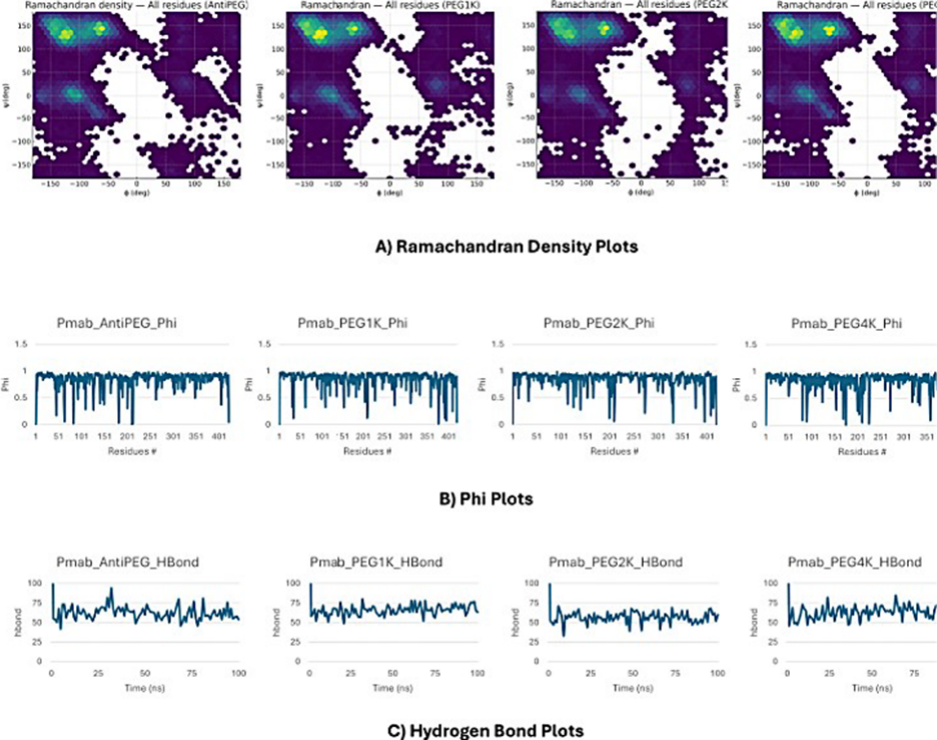
Dihedral and hydrogen bond analyses of PEGylated Fc constructs. (A) Ramachandran density plots show φ/ψ conformational sampling for AntiPEG, PEG1 k, PEG2 k, and PEG4 k variants. (B) Backbone φ-angle profiles reveal localized flexibility within the CH2–CH3 hinge region. (C) Hydrogen bond trajectories illustrate reduced interdomain hydrogen bonding frequency with increasing PEG size, supporting hinge expansion and partial destabilization of the Fc interface.

The 4 kDa construct’s elevated PC2 variance and circular vector distribution further indicate a multidimensional flexibility, possibly involving rotational freedom around the PEG linker, which may destabilize the Fc’s interaction with Fcγ receptors by altering the spatial orientation of key glycosylation sites. These findings, corroborated by RMSF data showing CH2 and CH3 mobility, underscore a PEG length-dependent modulation of Fc dynamics with implications for effector function and therapeutic efficacy that merit further experimental validation.

## Discussion

PEGylation of therapeutic antibodies remains a widely employed strategy to enhance pharmacokinetics, particularly circulation half-life, and reduce immunogenicity. However, the molecular consequences of PEG conjugation, especially on the Fc-region structure and dynamics, remain incompletely characterized. This study employed a multiscale modeling framework to dissect the effects of PEG linker length on the structural integrity and flexibility profile of Pertuzumab Fc constructs by using computational methods. Our findings reveal that PEGylation induces pronounced, length-dependent conformational changes within the Fc region, particularly affecting the CH2 and CH3 domains as well as hinge flexibility. These structural perturbations are likely to propagate allosterically through the antibody framework, potentially disrupting the spatial orientation of the Fab arms and complementarity-determining regions (CDRs). Such misalignment may impair efficient engagement with the HER2 receptor, contributing to the observed decrease in binding affinity with increasing PEG linker length. These findings suggest that PEGylation alters the structural dynamics of Fc, with consequences for biological function, as hinge flexibility and domain motion can impact HER2 receptor binding, Fcγ receptor interactions, and effector activity. The purpose of this study was to define how PEG linker length modulates antibody conformation in order to inform the design of PEGylated antibodies and antibody-nanoparticle conjugates for controlled pharmacokinetics and target engagement.

Initial structural validation showed that the models possessed high AlphaFold2 confidence metrics (pLDDT > 70, pTM > 0.7) and strong concordance with experimental templates from the PDB and EMDB. Glycosylated Asn297 residues were stably maintained throughout simulation, consistent with prior structural and functional studies emphasizing the importance of glycan integrity for Fc stability and effector function.^16,47,48^ Prior analyses indicate that N297 glycans stabilize the Fc dimer by increasing the energetic cost of both CH2 closure and opening; the hinge further constrains the opening via its disulfide linkage. In our PEGylated variants, increased hinge separation relative to the glycan-stabilized state indicates that the PEG chain shifts the ensemble toward more open CH2–CH3 orientations.^20^

The MD simulations reinforced these results by revealing increased RMSD and hinge separation in the longer PEGylated constructs. PCA further distinguished the nature of motion shifts, with anti-PEG and glycan control showing compact CH2 inward motions, while PEGylated systems exhibited expansion of CH3 domains and greater mobility near the PEG-glycan linkage. Since RMSD calculations included both glycans and PEG linkers, the elevated values partly reflect PEG mobility; however, the parallel backbone RMSF and PCA trends confirm that Fc domain flexibility is also genuinely enhanced by longer PEG chains. These results collectively describe domain-resolved dynamics within the Fcregion, showing how PEG linker length modulates the relative mobility of the CH2 and CH3. This shift in dominant fluctuation patterns is consistent with previous studies showing that glycan modifications and PEGylation can alter Fc dimer packing and allosteric communication across the antibody framework.^36,39^ Since PEGylation is increasingly applied in nanoparticle formulations, the observed linker-dependent Fc modulations highlight the need to balance steric shielding benefits with preservation of the structural orientation required for binding.

To further characterize the structural origin of these domain motions, residue-level backbone dihedral (φ/ψ) and hydrogen bond analyses were performed. Ramachandran distributions revealed broadened φ/ψ sampling localized to the CH2–CH3 hinge (residues 320–340) in PEGylated systems, indicating increased torsional freedom and partial hinge opening. Complementary hydrogen bond analysis demonstrated a reduction in interdomain hydrogen bond occupancy compared with the anti-PEG control, particularly within the CH2–CH3 interface. These findings corroborate the PCA-derived flexibility patterns and confirm that PEGylation weakens key stabilizing interactions, promoting progressive hinge expansion and multidimensional motion within the Fc region. Together, these analyses provide residue-level evidence that PEGylation-induced steric and entropic effects destabilize interdomain contacts and modulate Fc conformational equilibria.

Collectively, these results support the conclusion that PEGylation perturbs Fc structural dynamics in a manner dependent on PEG linker length, leading to increased flexibility and hinge opening that may disrupt receptor engagement. The observed allosteric effects highlight the sensitivity of Fc-mediated interactions to glycan modification and polymer conjugation. This study demonstrates the value of integrating deep learning-based structural prediction, chemically informed modeling, and multiscale molecular simulations to investigate the mechanistic basis of functional changes in antibody conjugates. Such an approach provides a scalable framework for optimizing PEGylation strategies in the rational design of antibody-based therapeutics.

## Conclusions

Building on these findings, this study offers a mechanistic understanding of how the PEG linker length modulates Fc structure and dynamics. The progressive increase in conformational flexibility and hinge separation with longer PEG chains, particularly in the 2 and 4 kDa system, was associated with reduced binding free energy and altered domain motions localized to the CH2 and CH3 regions. These observations align with experimental findings reporting decreased receptor affinity upon PEGylation, yet until now, structural explanations for these effects have remained largely inferential. By visualizing domain-specific displacements and steric perturbations through atomistic simulations and PCA, this study provides a structural rationale for the observed functional impairment. Furthermore, backbone dihedral and hydrogen bond analyses substantiated these findings by revealing localized torsional relaxation and loss of interdomain hydrogen bonding, offering atomistic evidence that PEG chain length directly governs hinge expansion and Fc destabilization. These computational models bridge a critical gap between empirical affinity measurements and the molecular mechanisms underlying them, offering visual and quantitative insight into how PEGylation disrupts Fc conformational integrity. Collectively, these results support the rational design of PEGylated monoclonal antibodies in therapeutic applications requiring precise control over the molecular orientation and receptor engagement. The mechanistic basis identified here is also applicable to PEGylated antibody-nanoparticle systems, where linker length must be optimized to extend pharmacokinetics while maintaining receptor binding and Fc-mediated function.

## Supplementary Material



## Data Availability

The input and output files for the AlphaFold2 prediction and RoseTTAFold2 refinement, along with the parameter files, input.gro files, output.pdb files, and md.mdp file used in the molecular dynamics simulations, are available through Zenodo DOI: 10.5281/zenodo.16787149.

## Notes

Additional molecular dynamics simulation results for anti-PEG control and PEGylated pertuzumab Fc variants, including PCA scree and score plots; RMSF per-residue fluctuation plots; hinge separation structural overlays; RMSD profiles; potential energy trajectories; temperature trajectories; and structural diagram of pertuzumab–HER2 domain II complex (PDF)
