# Supplementary material for: Multiscale Computational Studies of PEG Chain Length Effects on HER2 mAb Fc Structure and Binding Energetics
Source: ACS Omega. 2025 Dec 5;10(49):60688–96. doi: 10.1021/acsomega.5c08654 (PMC12713491; doi:10.1021/acsomega.5c08654)
Supplement: Supplementary file 1 [file ao5c08654_si_001.pdf]

Multiscale Computational Studies of PEG Chain Length Effects on HER2 mAb Fc Structure and Binding Energetics

Heather A. Noriega [1,2], Emmanuel O. Akala [2], Xiang Simon Wang[1,2*]

[1]Artificial Intelligence and Drug Discovery (AIDD) Core Laboratory for District of Columbia Center of AIDS Research (DC CFAR) Washington DC, United States, [2]Department of Pharmaceutical Sciences, College of Pharmacy, Howard University, Washington DC, United States

Corresponding Author

* Xiang Simon Wang
xiang.wang@Howard.edu
202-806-6547

ASSOCIATED CONTENT

Additional molecular dynamics simulation results for Anti-PEG control and PEGylated pertuzumab Fc variants, including: PCA scree and score plots; RMSF per-residue fluctuation plots; hinge separation structural overlays; RMSD profiles; potential energy trajectories; temperature trajectories; structural diagram of pertuzumab–HER2 domain II complex. This material is available free of charge via the Internet at http://pubs.acs.org.

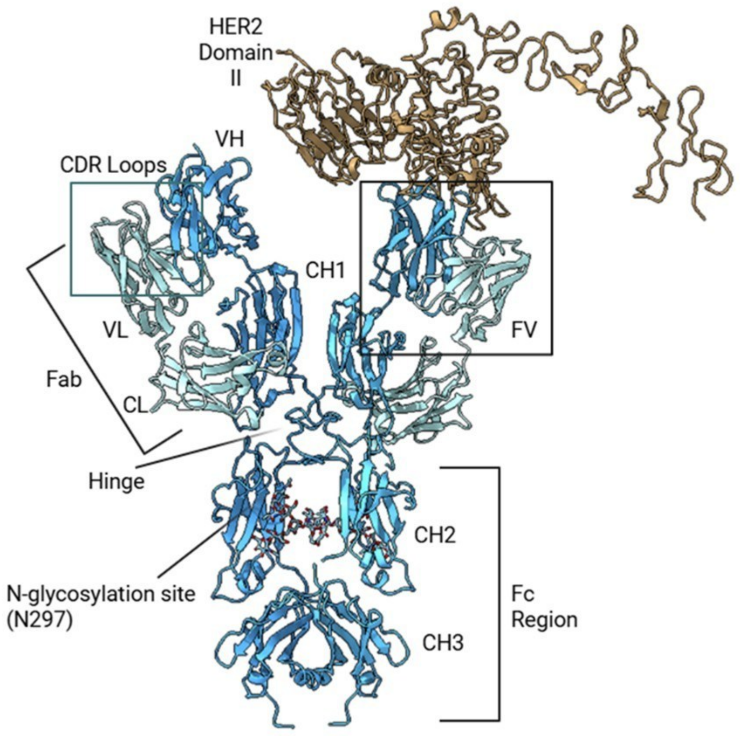

Figure S1: Structure of Pertuzumab bound to HER2 domain II. The IgG1 antibody Pertuzumab is shown in complex with HER2 domain II (brown). Key regions are labeled, including the Fab arms, CDR loops, hinge, Fc region (CH2/CH3), and the N-glycosylation site at N297. Pertuzumab binds HER2 at subdomain II to block receptor dimerization.

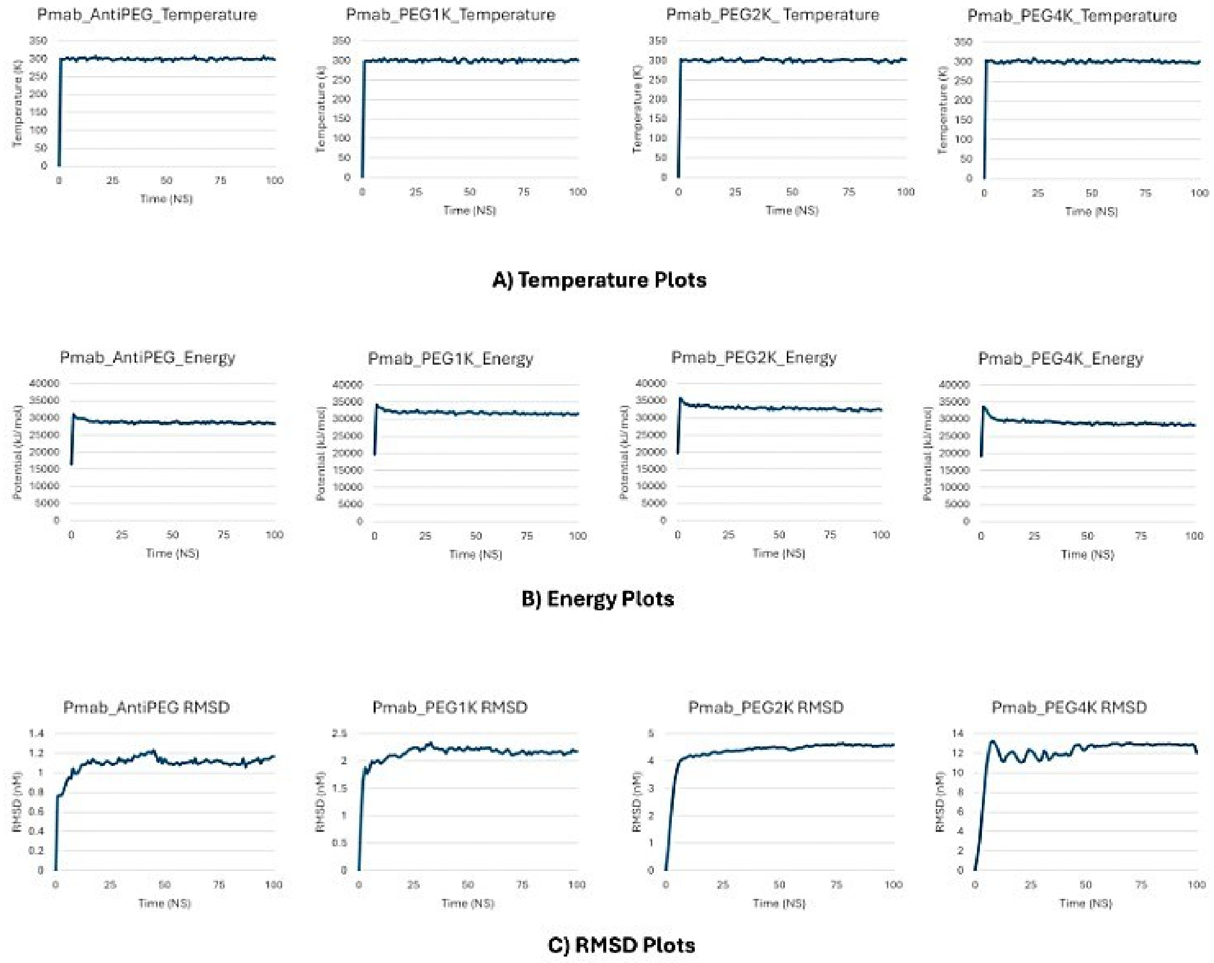

Figure S2: System equilibration profiles for non-PEGylated and PEGylated Pertuzumab Fc constructs. (A) *Temperature plots* show stable thermal equilibration near 300 K across all systems throughout 100 ns simulations. (B) *Energy plots* indicate rapid convergence of potential energy within the first few nanoseconds, confirming thermodynamic stability of each trajectory.(C) *RMSD plots* demonstrate initial structural relaxation within the first 10–20 ns, followed by stable fluctuations, with increasing RMSD magnitudes observed for longer PEG chains (1 kDa < 2 kDa < 4 kDa), consistent with enhanced conformational flexibility upon PEGylation.

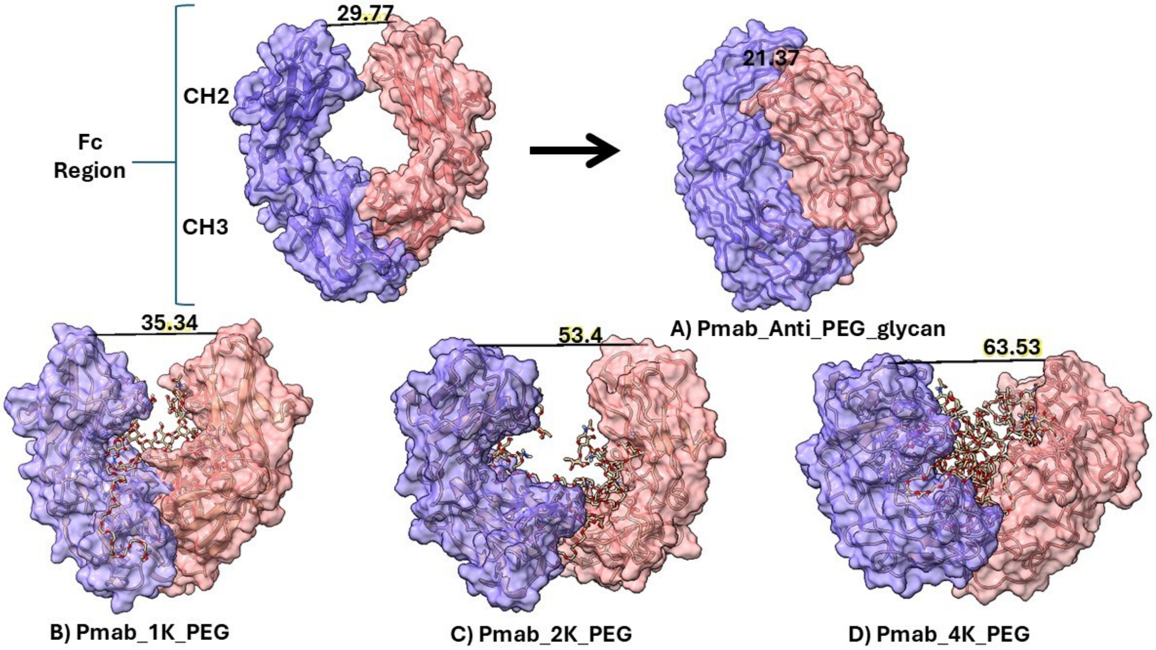

Figure S3: Structural dynamics of pertuzumab (Pmab) Fc region upon PEGylation. Molecular simulations illustrate the anti-PEGylated glycan control (A) with a hinge-proximal inter-residue distance decreasing from 29.77 Å to 21.37 Å, and PEGylated variants with distances of 35.34 Å (B, 1 kDa), 53.40 Å (C, 2 kDa), and 63.53 Å (D, 4 kDa)at the end of 100ns, highlighting progressive hinge expansion with increasing PEG linker length. The pink (heavy chain1) and purple (heavy chain 2) surfaces represent Fc heavy chains, respectively, corresponding to the CH2-CH3 domains of the Fc region shown in the start figure.

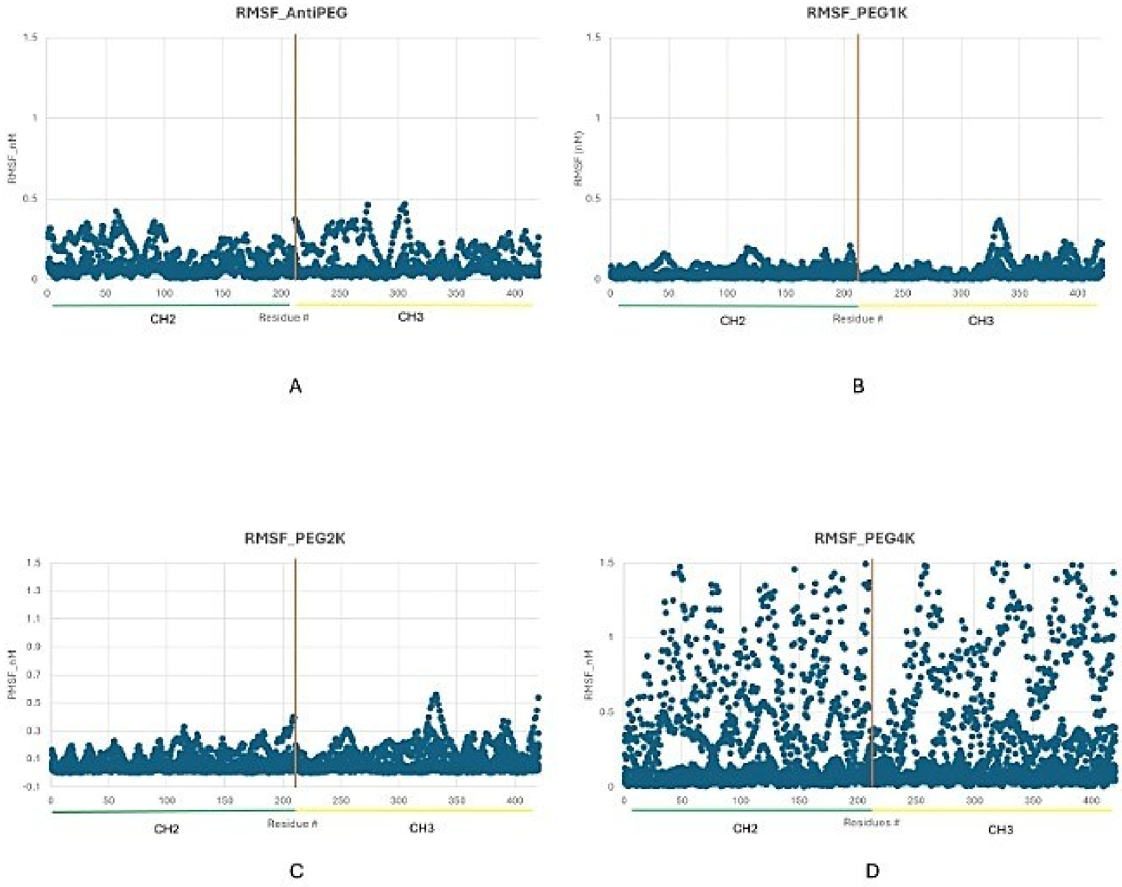

Figure S4: **Root mean square fluctuation profiles of the Fc region across PEGylated and non-PEGylated systems**. (A) Anti-PEG control, (B) PEG-1 kDa, (C) PEG-2 kDa, and (D) PEG-4 kDa. Each plot shows residue-wise flexibility (RMSF, Å) along the Fc domain, with CH2 and CH3 domains labeled below the x-axis and the orange vertical line denoting the hinge interface. Increasing PEG length corresponded to a progressive redistribution of flexibility from the CH2 region toward CH3, with the 4 kDa construct exhibiting the highest overall amplitude.

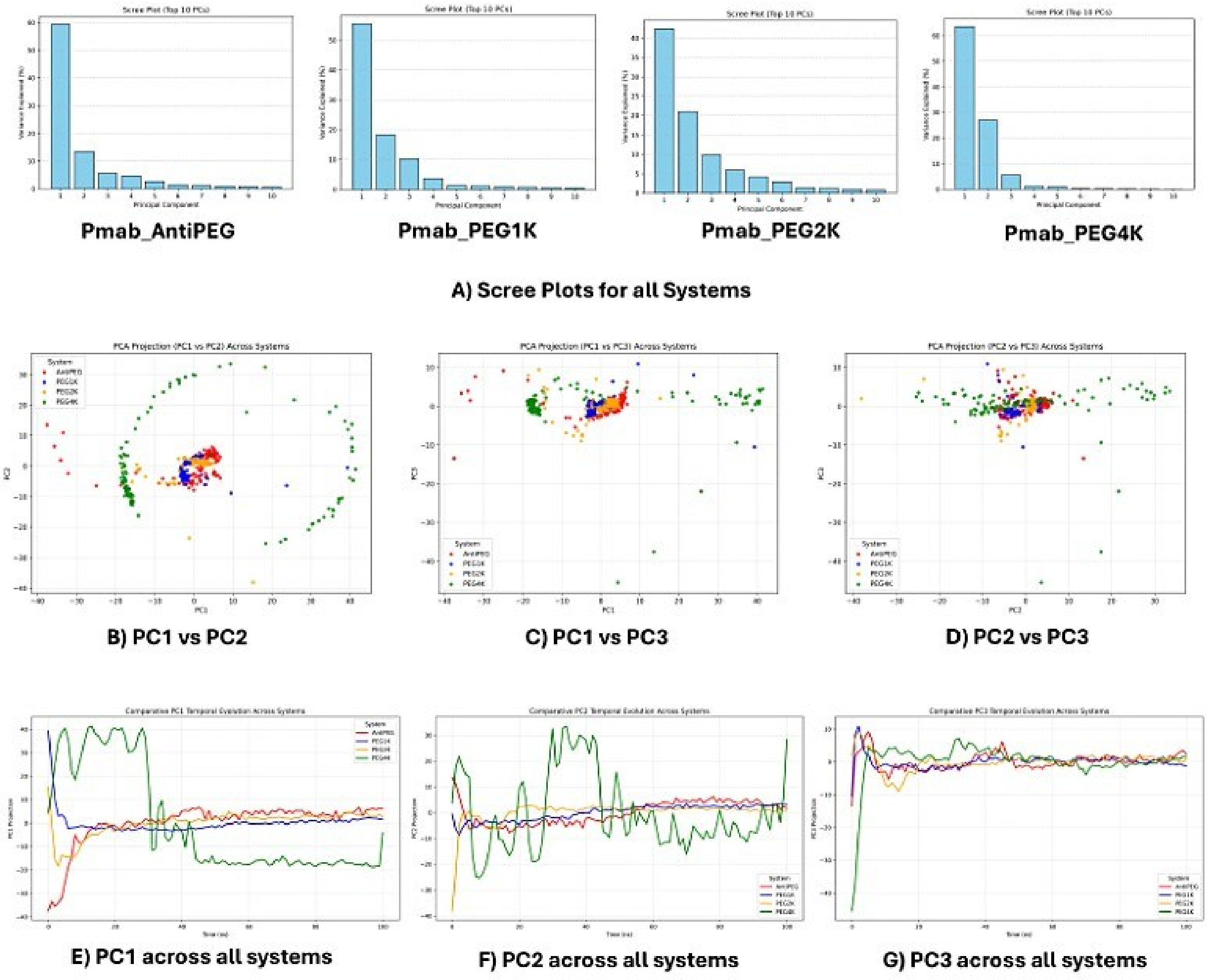

Figure S5: Principal component analysis (PCA) of pertuzumab (Pmab) Fc constructs. (A) Scree plots showing eigenvalue distribution for each system (AntiPEG, PEG1K, PEG2K, PEG4K). (B-D) 2D projections of principal components illustrating conformational clustering and motion along PC1-PC2, PC1-PC3, and PC2-PC3. (E-G) Temporal evolution of PC1, PC2, and PC3 across all systems.

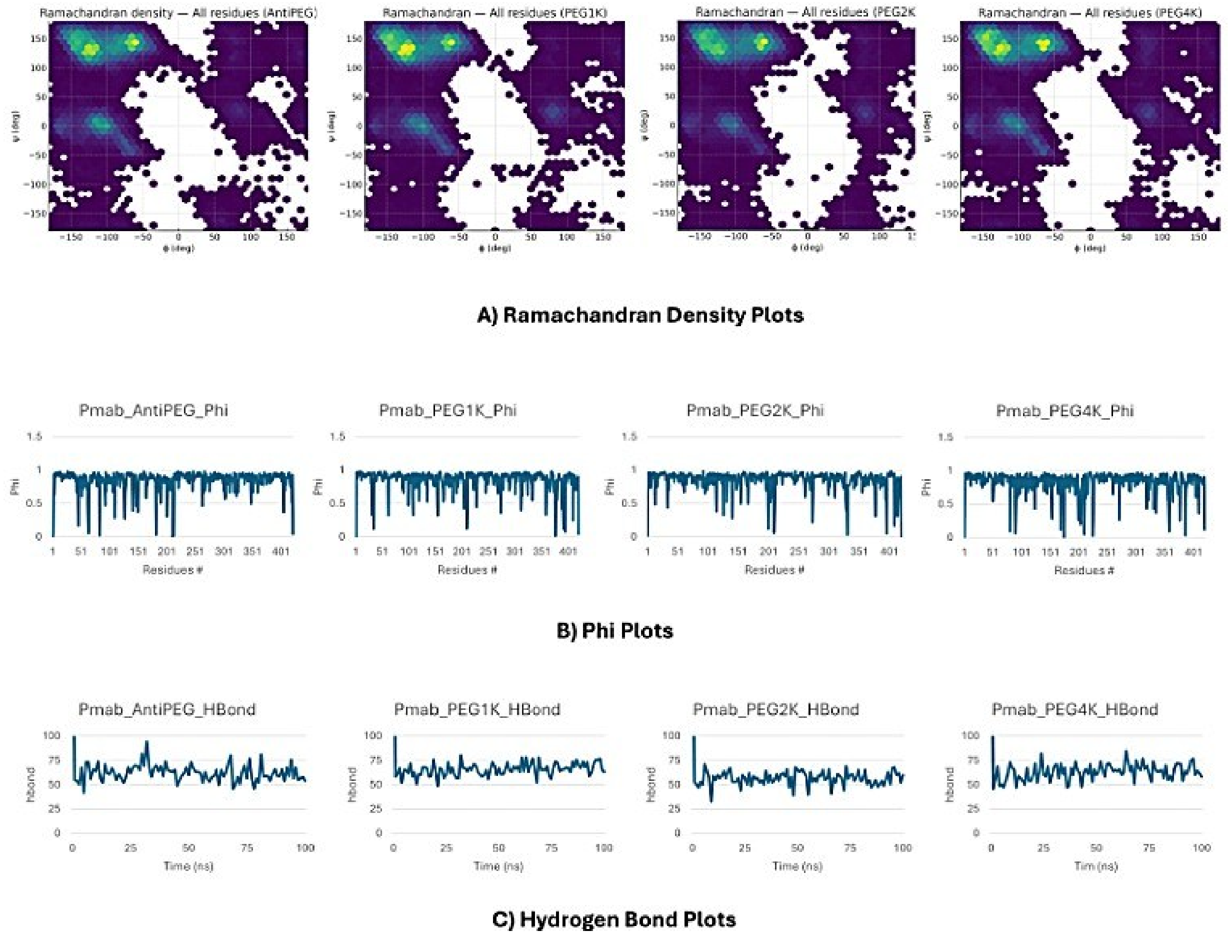

Figure S6: Dihedral and Hydrogen Bond Analyses of PEGylated Fc Constructs. (A) Ramachandran density plots show φ/ψ conformational sampling for AntiPEG, PEG1 k, PEG2 k, and PEG4 k variants. (B) Backbone φ-angle profiles reveal localized flexibility within the CH2–CH3 hinge region. (C) Hydrogen bond trajectories illustrate reduced inter-domain hydrogen bonding frequency with increasing PEG size, supporting hinge expansion and partial destabilization of the Fc interface.
